# ¿What do we know about Very Early Onset Schizophrenia? - A case report

**DOI:** 10.1192/j.eurpsy.2023.1527

**Published:** 2023-07-19

**Authors:** L. Unzue, J. Royo, L. Llovera, A. Corrales

**Affiliations:** 1 Child and adolescent psychiatry; 2 Psychiatry, Navarra’s University Hospital, Pamplona, Spain

## Abstract

**Introduction:**

This is a case aboout a 12-year-old girl, lives with her mother, father and younger brother. Parents originally from Peru, the patient was born in Spain.

She started follow-up by Mental Health in May 2022 due to low self-esteem after school bullying. The parents report that she has always been a shy child with many insecurities; she has always been insecure and suspicious in her relationships with peers, but during the last school year she was more nervous and insecure with everything that had happened to her; this situation was the reason for requesting a consultation at an Infant-Juvenile Mental Health Center when the school year was about to end.

In October she went to the emergency department accompanied by her parents because she seemed different in the last few days. A few days ago, while the patient was returning on the school bus, the patient began to write to her mother commenting that her classmates were imitating her, and that she noticed strangers not only in her classmates but also in her teachers, and in her own parents, thinking that they were imitating her. She had not slept well for several days.

In the interview alone with the patient, she was distrustful, hypervigilant, referring that the therapists were imitating her. She verbalized delusional ideation of harm and auditory hallucinations with high distress and emotional repercussions. She also presented slightly disorganized speech and formal thought disorders. In the last few days she had also been aggressive towards her younger brother and mother.

She had presented a febrile condition two weeks ago that subsided with antibiotics.

As a family history, she has a father’s aunt diagnosed with paranoid schizophrenia.

**Objectives:**

Due to the seriousness of the situation and the impossibility of performing an outpatient approach, it was decided to perform an urgent admission to the Infanto-Juvenile Brief Inpatient Unit with the objectives of performing a diagnostic affiliation, organic screening and evolutionary follow-up.

**Methods:**

Due to the febrile picture that had presented two weeks ago, and the recent onset of symptomatology, it was decided to request the following tests: abdominal ultrasound, cranial MRI and electroencephalogram. All tests were normal.

**Results:**

Due to the normality of the complementary tests requested, it was diagnosed as F29 Non-organic psychosis without specification. Treatment with Risperidone was started.

**Conclusions:**

Two months later, the patient has had a good evolution, but still continues to present psychotic symptoms, such as auditory hallucinations and delusional ideation of harm, but with less impact than at the beginning.

The evolution of the case points to a possible diagnosis of Schizophreniform Disorder, due to the persistence of psychotic symptoms, less than 6 months after the debut.

Due to the age of the patient, we would be facing a diagnosis of VEOS (Very Early Onset Schizophrenia).

**Disclosure of Interest:**

None Declared

